# Potential Anti-Inflammatory Effects of Gintonin-Enriched Fraction in TNF-α-Stimulated Keratinocytes

**DOI:** 10.3390/ijms262411864

**Published:** 2025-12-09

**Authors:** Rami Lee, Kyung-Jong Won, Ji-Hun Kim, Sun-Hye Choi, Sung-Hee Hwang, Seung-Yeol Nah

**Affiliations:** 1 Ginsentology Research Laboratory, Department of Physiology, College of Veterinary Medicine, Konkuk University, Seoul 05029, Republic of Korea; rmlee12@konkuk.ac.kr (R.L.); bioskjh@konkuk.ac.kr (J.-H.K.); 2 Department of Physiology and Premedical Science, College of Medicine, Konkuk University, Chungju 27478, Republic of Korea; kjwon@kku.ac.kr; 3 Department of Animal Health, College of Health and Medical Services, Osan University, Osan 18119, Republic of Korea; vettman@osan.ac.kr; 4 Department of Pharmaceutical Engineering, College of Health Sciences, Sangji University, Wonju 26339, Republic of Korea

**Keywords:** gintonin-enriched fraction, tumor necrosis factor-α, keratinocytes, skin, inflammation

## Abstract

Gintonin-enriched fraction (GEF), a bioactive glycolipoprotein derived from *Panax ginseng* is known for its potential as a natural anti-inflammatory agent. Keratinocytes are closely related to the development and progression of various inflammatory skin conditions. However, the effect of GEF on inflammation-related responses in keratinocytes remains unclear. This study aimed to investigate whether GEF modulates key inflammatory responses in keratinocytes stimulated by tumor necrosis factor (TNF)-α. The effect of GEF on biological activities in TNF-α-stimulated keratinocytes (HaCaT cells) was evaluated using water-soluble tetrazolium salt, enzyme-linked immunosorbent, immunostaining, and immunoblotting assays. In TNF-α-stimulated HaCaT cells, GEF attenuated reactive oxygen species production, nitric oxide release, and inducible nitric oxide synthase expression. Moreover, GEF reduced the release of interleukin (IL)-6 and RANTES, while increasing the release of IL-10 in TNF-α-exposed HaCaT cells. Additionally, GEF treatment resulted in reduced cyclooxygenase-2 expression and prostaglandin E_2_ release and inhibited TNF-α-induced translocation of nuclear factor-κB in HaCaT cells. Furthermore, TNF-α and IL-6 levels in ultraviolet B-irradiated HaCaT cells were reduced by GEF treatment. These findings indicated that GEF exerts anti-inflammatory effects on keratinocytes. This study provides a basis for the development of novel therapeutic approaches for the prevention and treatment of inflammatory skin disorders.

## 1. Introduction

Inflammation is a vital biological defense process; it helps the body respond to infections, injuries, or damaging agents by initiating healing and restoring tissue balance [1,2]. It involves a complex cascade of molecular and cellular events characterized by the activation of various immune cells and the release of inflammatory mediators [1,3]. Inflammation is essential for protecting the body; however, its dysregulation can lead to chronic inflammatory conditions [2,3]. These conditions are associated with the development of various diseases, including autoimmune disorders, cancer, neurodegenerative diseases, and skin disorders [1,4]. Excessive or prolonged inflammation in the skin is a hallmark of dermatological diseases such as psoriasis and atopic dermatitis [5]. Many inflammatory responses in the skin are mediated by keratinocytes, which are the major cell type in the epidermis. These cells serve as the first line of defense against environmental stressors and are capable of initiating and amplifying inflammatory reactions through the production of various mediators [6].

Keratinocytes play a pivotal role in the inflammatory response of the skin, and their abnormal activation can contribute to the pathogenesis of various inflammatory skin conditions [7]. Inflammation in keratinocytes is modulated through various signaling pathways, including the mitogen-activated protein kinases (MAPKs) and the nuclear factor-κB(NF-κB) pathways [8,9]. These cells produce reactive oxygen species (ROS) upon exposure to harmful external stimuli. ROS participate in the inflammatory cascade by inducing various inflammation-related factors such as pro-inflammatory cytokines, chemokines, and other mediators that recruit and activate immune cells [9,10,11]. Among the key pro-inflammatory cytokines released by keratinocytes are interleukin (IL)-6, tumor necrosis factor-alpha (TNF-α), and RANTES (regulated upon activation, normal T cell expressed and secreted)/CCL5 [chemokine (c-c motif) ligand 5], all of which are known to contribute to the persistence and amplification of inflammation [12,13,14]. Also, cyclooxygenase-2 (COX-2) is a critical enzyme involved in the synthesis of pro-inflammatory prostaglandins, which further exacerbate the inflammatory process [15]. The production of nitric oxide (NO), a potent signaling molecule, is another critical feature of inflammation [16]. NO is synthesized by inducible nitric oxide synthase (iNOS) in response to pro-inflammatory stimuli and has been shown to play a dual role in inflammation, contributing to both protective and pathological effects, depending on its concentration and timing of release [16,17].

A balance between pro-inflammatory and anti-inflammatory signals is crucial for resolving inflammation [3]. For example, the cytokine IL-10 is a key anti-inflammatory mediator that plays a crucial role in resolving inflammation and promoting tissue repair [18]. IL-10 exerts its effects by suppressing the production of pro-inflammatory cytokines in inflammation-related cells, such as immune cells and keratinocytes, and by inhibiting the activation of immune cells, thereby preventing excessive tissue damage [18]. In contrast, chronic inflammation of the skin can lead to a breakdown of this balance, resulting in persistent tissue injury and development of inflammatory skin diseases [3]. Therefore, understanding the molecular mechanisms that regulate the inflammatory response in keratinocytes is crucial for developing effective therapeutic strategies for inflammatory skin conditions.

Recently, natural products have garnered significant attention because of their potential to modulate inflammatory pathways [19,20]. One such compound is the gintonin-enriched fraction (GEF) derived from the roots of *Panax ginseng* C. A. Meyer (*P. ginseng*), which is renowned for its medicinal properties in traditional medicine [21,22]. It is composed of lipids (20.2%), amino acids (30.3%), and carbohydrates (30%) [21]. GEF contains bioactive lipids, such as lysophosphatidic acid (LPA), phosphatidic acids, and fatty acids. LPA, known as the primary active constituent of gintonin, is also regarded as the major bioactive component responsible for the effect of GEF [21,22].

GEF possesses a range of bioactive effects, including anti-inflammatory, antioxidant, anticancer, and neuroprotective properties [21,23,24]. Multiple studies have shown that GEF modulates immune responses by inhibiting the expression of pro-inflammatory mediators [21,25]. These findings suggest that GEF is a promising candidate for the treatment of inflammation-related skin diseases. However, the potential of GEF in regulating inflammatory responses in keratinocytes remains poorly understood and warrants further investigation. Therefore, in this study, we aimed to investigate the effects of GEF on key inflammation-related mediators in TNF-α-stimulated HaCaT cells. We also evaluated its potential as a therapeutic agent for inflammatory skin disorders.

## 2. Results

### 2.1. Effect of GEF and TNF-α on Cell Viability of HaCaT Cells

**Figure 1:**
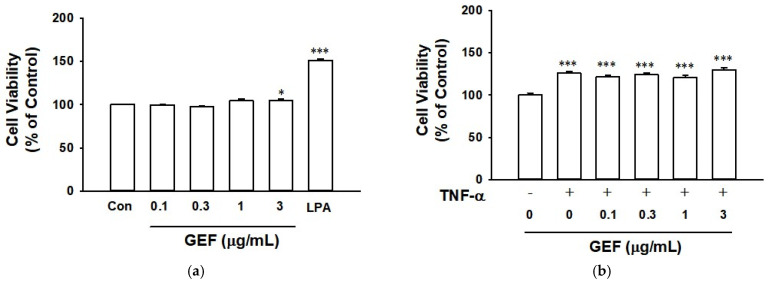
Effect of TNF-α and gintonin-enriched fraction (GEF) on HaCaT cell viability. (**a**) Cell viability by GEF. Cells were incubated with or without GEF at the indicated concentrations for 24 h. Cells were subjected to a water-soluble tetrazolium formazan (WST)-based assay. Lysophosphatidic acid (LPA; 10 µM) was used as a positive control. (**b**) Cell viability by TNF-α in the presence of GEF. Cells were pretreated with GEF at the indicated concentration for 1 h and incubated with or without 10 ng/mL of TNF-α for 24 h. Cells were subjected to a WST-based assay. The response of the untreated cell was counted as 100%. Data are presented as means ± S.E.M. (*n* = 6, (**a**); *n* = 6, (**b**)); * *p* < 0.05, *** *p* < 0.001 vs. untreated control cells. Con, untreated control. TNF-α, tumor necrosis factor-α.

To determine the non-toxic concentrations of GEF in keratinocytes, we tested the effect of GEF on human skin keratinocytes (HaCaT cells) using a water-soluble tetrazolium (WST)-based assay. GEF showed no effect on HaCaT cell viability at concentrations of 0.1 to 1 µg/mL and induced a slight increase in cell viability at a concentration of 3 µg/mL (Figure 1a). Positive control LPA (10 µM) significantly increases cell viability of HaCaT cells. To determine the non-toxic concentrations of GEF on TNF-α-stimulated HaCaT cells, we pretreated HaCaT cells with GEF (0.1–3 µg/mL) followed by treatment with TNF-α (10 ng/mL) and HaCaT cell viability was evaluated using a WST analysis. Treatment with TNF-α (10 ng/mL) for 24 h significantly increased the viability of HaCaT cells by an approximate 30%. The TNF-α-increased cell viability was not affected in the presence of GEF at concentrations ranging from 0.1 to 1 µg/mL but was slightly, although not significantly, increased in the presence of 3 µg/mL GEF (Figure 1b). These results led us to use GEF concentrations up to 1 µg/mL in further experiments in this study.

### 2.2. Effect of GEF on TNF-α-Induced ROS Production in HaCaT Cells

**Figure 2:**
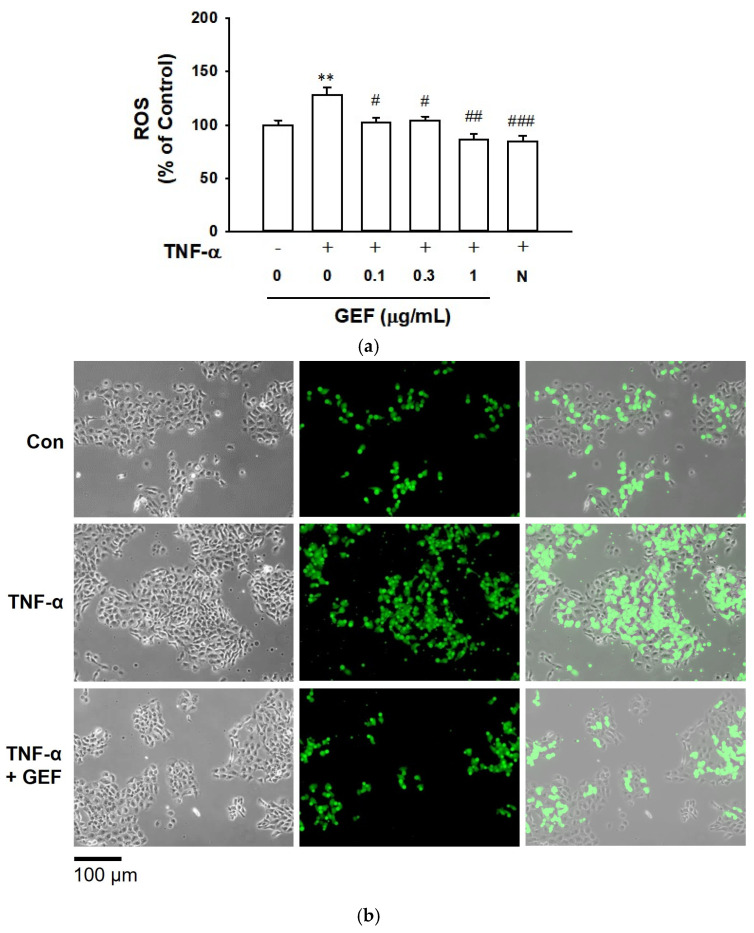
Effect of gintonin-enriched fraction (GEF) on TNF-α-induced reactive oxygen species (ROS) production in HaCaT cells. Cells were pretreated with GEF at the indicated concentrations for 1 h and incubated with or without 10 ng/mL of TNF-α for 40 min. TNF-α-stimulated ROS production in cells was analyzed using the cell-permeable fluorogenic probe (CM-H2DCFDA) for 30 min, according to the manufacturer’s instructions. (**a**) Quantitative analysis of the intensity of fluorescence correlated with ROS production. N-acetyl L-cysteine (N) was used as a positive control. The response of the untreated cell was counted as 100%. Data are presented as means ± S.E.M. (*n* = 6); ** *p* < 0.01 vs. untreated control cells; ^#^ *p* < 0.05, ^##^ *p* < 0.01, ^###^ *p* < 0.001 vs. TNF-α alone. (**b**) Representative images of CM-H2DCFDA-treated cells. Cells were pretreated with 1 µg/mL of GEF for 1 h and incubated with or without 10 ng/mL of TNF-α for 2 h. Cells were incubated with CM-H2DCFDA as in Section 4. Green color indicates CM-H2DCFDA-positive cells. Scale bar: 100 µm. Con, untreated control. TNF-α, tumor necrosis factor-α.

To examine the inhibitory effect of GEF on TNF-α-induced oxidative stress in HaCaT cells [11], we measured GEF-altered level of ROS in TNF-α-exposed HaCaT cells. TNF-α (10 ng/mL) significantly increases the ROS production. Treatment with GEF (0.1–1 µg/mL) significantly reduced the TNF-α-induced ROS production, as shown in Figure 2a. This effect of GEF (1 µg/mL) was similar to that of N-acetyl-L-cysteine (1 mM), as commonly known as a ROS scavenger [26]. Additionally, fluorescence images also supported the TNF-α-induced ROS production and inhibitory effect of GEF (1 µg/mL) on TNF-α-induced ROS production (Figure 2b).

### 2.3. Effect of GEF on TNF-α-Induced NO Release in HaCaT Cells

**Figure 3:**
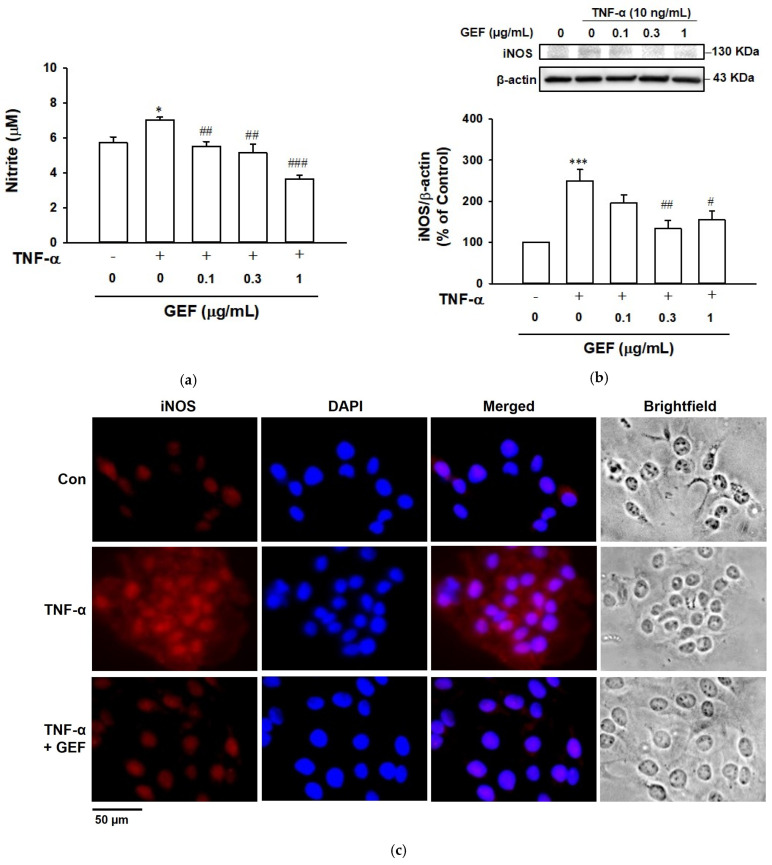
Effect of gintonin-enriched fraction (GEF) on TNF-α-induced nitric oxide (NO) release and inducible NO synthase (iNOS) expression in HaCaT cells. (**a**) NO level. Cells were pretreated with GEF at the indicated concentrations for 1 h and incubated with or without 10 ng/mL of TNF-α for 24 h. TNF-α-stimulated NO level in cell culture supernatant was analyzed using Griess reagent. (**b**) iNOS expression resulted from immunoblotting. Cells were pretreated with GEF for 1 h at the indicated concentration and incubated with or without 10 ng/mL of TNF-α for 24 h. Cell lysates in each test were immunoblotted using the corresponding antibodies. Each upper panel shows a representative image of immunoblotting. Lower graphs represent the results expressed as percentages of untreated control cells (control). Data are presented as means ± S.E.M. (*n* = 4–6, (**a**); *n* = 4, (**b**)); * *p* < 0.05, *** *p* < 0.001 vs. untreated control cells; ^#^ *p* < 0.05, ^##^ *p* < 0.01, ^###^ *p* < 0.001 vs. TNF-α alone. (**c**) Representative images of iNOS-immunostained cells. Cells were pretreated with 1 µg/mL of GEF for 1 h and incubated with or without 10 ng/mL of TNF-α for 24 h. Cells were immunostained using the corresponding antibody as in Section 4. Red and blue indicate iNOS- and DAPI-positive cells, respectively. Scale bar: 50 μm. Con: untreated control. TNF-α, tumor necrosis factor-α.

To analyze the effect of GEF on NO production in keratinocytes, we initially assessed the total nitrite content, a well-known indicator of NO release in HaCaT cells [27]. Total nitrite levels were quantified in the supernatant (conditioned medium) of the culture medium in which cells were cultured under specific test conditions. In untreated control cells, the total released nitrite content was 5.71 ± 0.34 µM (Figure 3a). After incubation with TNF-α (10 ng/mL), the total nitrite was significantly increased to 7.03 ± 0.26 µM. GEF (0.1–1 µg/mL) significantly reduced TNF-α-induced nitrite release in a concentration-dependent manner, with the maximum effect at a concentration of 1 µg/mL (3.63 ± 0.25 µM) (Figure 3a).

We also analyzed the effect of GEF on TNF-α-induced iNOS expression in HaCaT cells using immunoblotting and immunostaining analyses. iNOS expression was increased by TNF-α (10 ng/mL) treatment in both analyses. In immunoblotting analysis, GEF at 0.3 and 1 µg/mL significantly reduced the TNF-α-increased iNOS expression in HaCaT cells (Figure 3b). Similarly to this result, immunostaining analysis revealed that TNF-α-increased iNOS expression was attenuated by treatment with GEF at 1 µg/mL (Figure 3c). These results indicated that GEF may decrease NO production by reducing iNOS expression in TNF-α-treated HaCaT cells.

### 2.4. Effect of GEF on TNF-α-Induced Cytokine Release in HaCaT Cells

**Figure 4:**
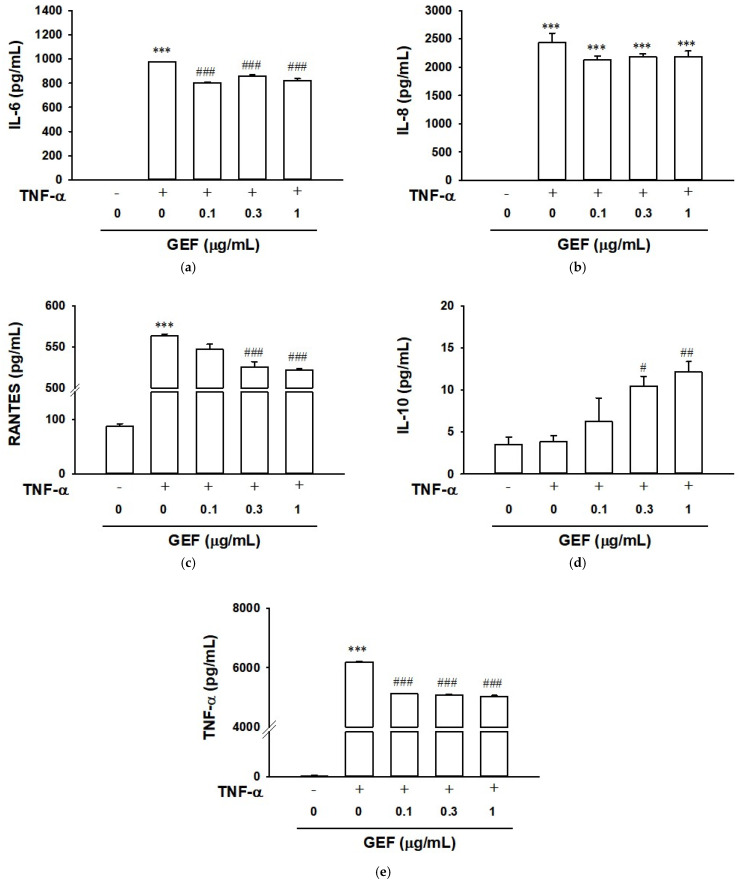
Effect of gintonin-enriched fraction (GEF) on TNF-α-induced cytokine release in HaCaT cells. Cells were pretreated with GEF at the indicated concentrations for 1 h and incubated with or without 10 ng/mL of TNF-α for 24 h. TNF-α-stimulated cytokine release in conditioned medium was analyzed by an enzyme-linked immunosorbent assay (ELISA) as in Section 4. (**a**) IL-6; (**b**) IL-8; (**c**) RANTES; (**d**) IL-10; (**e**) TNF-α. Data are presented as means ± S.E.M. (*n* = 6); *** *p* < 0.001 vs. untreated control cells; ^#^ *p* < 0.05, ^##^ *p* < 0.01, ^###^ *p* < 0.001 vs. TNF-α alone. IL-6, interleukin-6; IL-8, interleukin-8; IL-10, interleukin-10; RANTES, regulated on activation, normal T-cell expressed and secreted/CCL5 [chemokine (c-c motif) ligand 5]; TNF-α, tumor necrosis factor-α.

To evaluate the effect of GEF on cytokines release in keratinocytes, cytokine levels were measured in the supernatant (conditioned medium) of culture medium in which HaCaT cells were cultured in the presence or absence of TNF-α with or without GEF. TNF-α (10 ng/mL) significantly increased the levels of pro-inflammatory cytokines IL-6 (Figure 4a), IL-8 (Figure 4b), RANTES (Figure 4c), and TNF-α (Figure 4e). GEF reduced the TNF-α-enhanced release levels of cytokines, IL-6, and RANTES at concentrations of 0.1 to 1 µg/mL (Figure 4a,c). GEF (0.1–1 µg/mL) slightly, but not significantly, decreased the level of IL-8 release enhanced by TNF-α (Figure 4b). On the other hand, TNF-α (10 ng/mL) did not increase the level of an anti-inflammatory cytokine IL-10 release in HaCaT cells, but addition of GEF (0.3 and 1 µg/mL) increased IL-10 release level in TNF-α (10 ng/mL)-exposed HaCaT cells (Figure 4d). These results indicated that GEF may exert an anti-inflammatory effect.

### 2.5. Effect of GEF on TNF-α-Induced COX-2 and Prostaglandin E_2_ Expression in HaCaT Cells

**Figure 5:**
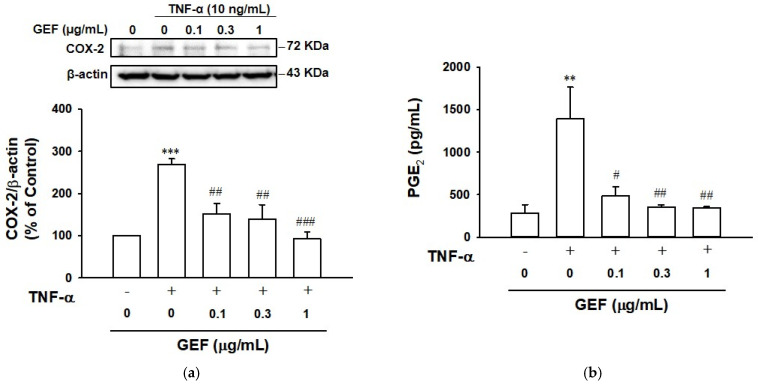
Effect of gintonin-enriched fraction (GEF) on tumor necrosis factor (TNF)-α-induced COX-2 expression and prostaglandin E_2_ (PGE_2_) release in HaCaT cells. (**a**) Cyclooxygenase-2 (COX-2) expression. Cells were serum-starved for 4 h and then incubated with serum-free medium containing GEF (0.1–1 μg/mL) for 1 h. Then the cells were incubated with or without 10 ng/mL of TNF-α for 24 h. Cell lysates in each test were immunoblotted using the corresponding antibodies. Upper panels show the representative images of immunoblotting. Lower statistical graphs represent results expressed as a percentage of untreated control cells. (**b**) PGE_2_ release. Cells were pretreated with GEF at the indicated concentrations for 1 h and incubated with or without 10 ng/mL of TNF-α for 24 h. TNF-α-stimulated PGE_2_ release level in cell-conditioned medium was analyzed by an enzyme-linked immunosorbent assay as in Section 4. Data are presented as means ± S.E.M. (*n* = 4 for (**a**), *n* = 3 for (**b**)); ** *p* < 0.01, *** *p* < 0.001 vs. untreated control cells; ^#^ *p* < 0.05, ^##^ *p* < 0.01, ^###^ *p* < 0.001 vs. TNF-α alone.

COX-2 participates in inflammation as a regulatory enzyme that induces the production of prostaglandin E_2_ (PGE_2_), an endogenous lipid mediator of inflammation [28]. To assess the effects of GEF on COX-2 in TNF-α-stimulated keratinocytes, we examined COX-2 expression in HaCaT cells pretreated with GEF and subsequently treated with or without TNF-α (10 ng/mL) using immunoblotting. As shown in Figure 5a, COX-2 expression was more highly expressed in TNF-α-treated HaCaT cells compared to untreated control cells. TNF-α-increased COX-2 expression in HaCaT cells was significantly reduced by the GEF (0.1–1 µg/mL) treatment.

To determine whether PGE_2_ is linked to the inhibitory effect of GEF on TNF-α-induced COX-2 expression in keratinocytes, we analyzed the effect of GEF on PGE_2_ release from TNF-α-stimulated HaCaT cells by an enzyme-linked immunosorbent assay (ELISA). PGE_2_ release was increased in TNF-α-treated HaCaT cells compared to untreated control cells. TNF-α-increased PGE_2_ release was significantly reduced by treatment with GEF at 0.1–1 µg/mL (Figure 5b).

### 2.6. Effect of GEF on TNF-α-Induced Translocation of NF-κB in HaCaT Cells

**Figure 6:**
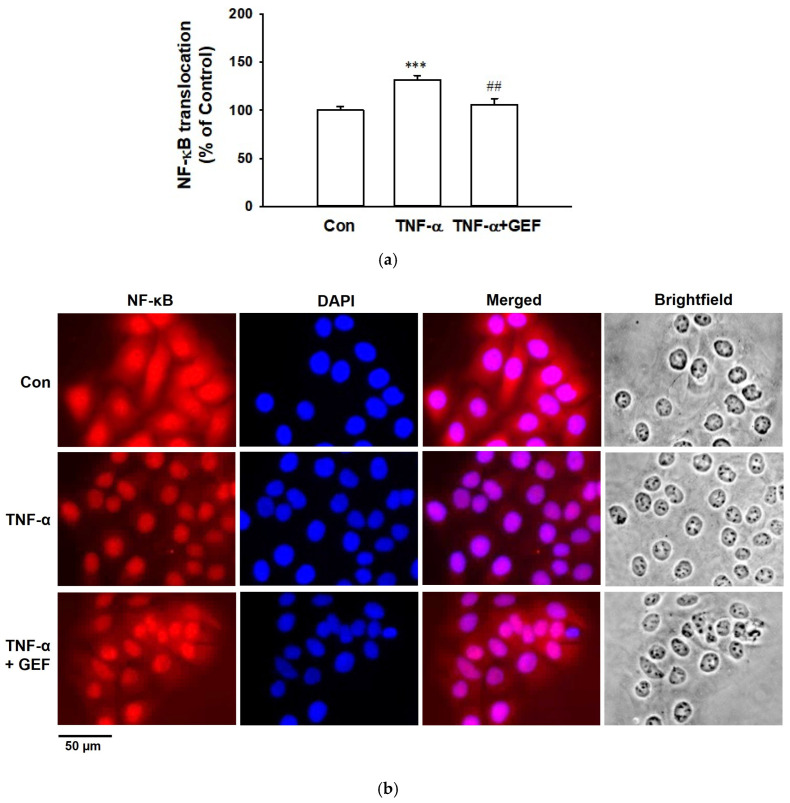
Effect of gintonin-enriched fraction (GEF) on translocation of NF-κB in HaCaT cells. Cells were pretreated with 1 µg/mL of GEF for 1 h and incubated with or without 10 ng/mL of TNF-α for 30 min. Cells were immunostained using corresponding antibody as in Section 4. (**a**) Quantification of NF-κB nuclear translocation. Nuclear-to-cytosolic fluorescence intensities were quantified using imageJ 1.54g (National Institutes of Health, Bethesda, MD, USA; http://imagej.org, Java 1.8.0_345). Data are presented as percentages relative to untreated control cells (Con). Data are presented as means ± S.E.M. (*n* = 8); *** *p* < 0.001 vs. untreated control cells; ^##^ *p* < 0.01 vs. TNF-α alone. (**b**) Fluorescence images of NF-κB-immunostained cells. Red and blue colors indicate NF-κB and DAPI positive cells, respectively. Scale bar: 50 μm. Con, untreated control cells; TNF-α, tumor necrosis factor-α.

NF-κB, a transcription factor, is closely associated with TNF-α-induced inflammatory response in keratinocytes and translocates into the nucleus to induce NF-κB-dependent inflammatory gene expression, linked to inflammation progression [29]. To examine whether GEF affects NF-κB in keratinocytes, we incubated HaCaT cells with or without TNF-α (10 ng/mL) in the presence of 1 µg/mL of GEF. Then, the translocation of NF-κB was analyzed using immunostaining technique. In untreated control cells, NF-κB was primarily localized in the cytoplasm, whereas TNF-α treatment induced its translocation to the nucleus in most HaCaT cells (Figure 6). However, in cells treated with both TNF-α and GEF, NF-κB was detected in both the cytoplasm and nucleus, indicating that GEF partially inhibited TNF-α-induced nuclear translocation of NF-κB.

### 2.7. Effect of GEF on Cytokine Release in Ultraviolet-Irradiated HaCaT Cells

**Figure 7:**
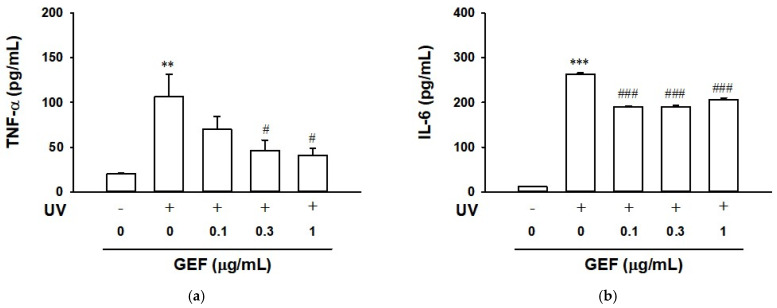
Effect of gintonin-enriched fraction (GEF) on cytokine release in ultraviolet irradiated HaCaT cells. Cells were exposed to ultraviolet B (UVB) radiation (10 mJ/cm^2^) and incubated with GEF at the indicated concentrations for 24 h. Then, cell culture media were centrifuged and cytokine release in conditioned medium was analyzed by an enzyme-linked immunosorbent assay (ELISA) as in Section 4. (**a**) TNF-α; (**b**) IL-6. Data are presented as means ± S.E.M. (*n* = 3–6); ** *p* < 0.01, *** *p* < 0.001 vs. untreated control cells; ^#^ *p* < 0.05, ^###^ *p* < 0.001 vs. UVB irradiated cells without GEF. TNF-α, tumor necrosis factor-α; IL-6, interleukin-6.

Ultraviolet B (UVB) is known to induce an inflammatory response in skin cells, and keratinocytes exposed to UVB can produce pro-inflammatory cytokines [15,30]. To observe the effect of GEF on pro-inflammatory cytokine TNF-α and IL-6 release in UVB-irradiated HaCaT cells, we analyzed cytokine release alteration using an ELISA. Cells were exposed to UVB irradiation (10 mJ/cm^2^) and then incubated with GEF for 24 h. UVB irradiation increased TNF-α levels (106.2 ± 25.2 pg/mL) compared to non-UVB irradiated control cells (19.9 ± 0.9 pg/mL). Treatment with GEF (1 µg/mL) significantly inhibited the UVB-induced increase in TNF-α release by approximately 61% (Figure 7a). As shown in Figure 7b, UVB irradiation increased IL-6 levels in the absence of GEF (262.8 ± 2.8 pg/mL) compared to non-UVB irradiated control cells (11.2 ± 0.4 pg/mL). Treatment with GEF (0.1–1 µg/mL) significantly inhibited the UVB-induced increase in IL-6 release by 22–28%. UVB irradiation (10 mJ/cm^2^) reduced HaCaT cell viability (53.3 ± 1.0%) and this was not affected by treatment with GEF at 0.1–1 µg/mL.

## 3. Discussion

In a previous study, the Korean red ginseng compound, gintonin, showed anti-inflammatory effects on mouse macrophage Raw264.7 cells [31]. Gintonin suppresses the production of NO and pro-inflammatory cytokines in macrophages [31]. Moreover, ginseng polysaccharide fraction, GEF, and crude ginseng saponin fraction as well as gintonin also showed anti-inflammatory responses by reducing nitric oxide production, phagocytosis, and release of IL-6 and TNF-α in the macrophage cells [21]. Gintonin also stimulates cell proliferation and migration, and the production of hairpin-binding epidermal growth factor-like growth factor and vascular endothelial growth factor in skin keratinocytes (HaCaT) [32,33]. These actions of gintonin may be associated with its skin wound-healing effects in skin diseases. Inflammatory skin diseases, such as psoriasis and atopic dermatitis, are associated not only with immune cells, such as macrophages, but also with skin cells [8,34,35]. However, the anti-inflammatory effects of gintonin and GEF on human keratinocytes have not been studied. Therefore, in this study we explored the effects of GEF on inflammatory responses in skin keratinocytes HaCaT exposed to TNF-α.

When keratinocytes are exposed to various environmental insults, such as pathogens, ultraviolet rays, and allergens, the cells produce inflammatory mediators such as cytokines, and dysregulation of these inflammatory mediators can lead to skin inflammation [7]. Excessive production of TNF-α has been shown to induce various inflammatory events in keratinocytes associated with the pathogenesis of inflammatory skin diseases [13,36]. Thus, TNF-α-exposed keratinocytes have been widely used as in vitro inflammatory models [13]. Because ROS act as inflammatory mediators, and antioxidants usually show anti-inflammatory effects [11], the ROS-scavenging activity of GEF was evaluated in the present study. GEF (0.1–1 µg/mL) did not affect the cell viability of TNF-α-treated HaCaT cells (Figure 1). GEF at this concentration range significantly reduced the TNF-α-induced ROS production (Figure 2), implying the potential of GEF as an antioxidant. In this study, we found that TNF-α induced production of NO in HaCaT cells (Figure 3a). These responses were accompanied by enhanced expression of iNOS (Figure 3b), COX-2 (Figure 5a), and PGE_2_ (Figure 5b). GEF treatment attenuated these responses induced by TNF-α (Figure 5). NO is synthesized by a family of three NO synthases (NOS): neuronal, inducible, and endothelial [17]. Inducible NOS (iNOS) is the major isoform expressed in most cells after induction by immunological or inflammatory stimuli [17]. In HaCaT keratinocytes, iNOS induction is dependent on inflammatory cytokines [37,38]. Therefore, our results indicate that GEF treatment may exert anti-inflammatory effects in keratinocytes by inhibiting iNOS expression and NO production.

IL-6 is produced by normal human keratinocytes in dermatological diseases and is involved in various responses, such as wound healing, inflammation, cell growth, and differentiation [12]. IL-6 produced by keratinocytes can promote the proliferation and migration of keratinocytes, affecting skin fibroblasts and leading to wound repair [39,40,41]. IL-6 deficiency can also lead to delayed wound closure. IL-6 is upregulated in inflammatory skin conditions such as psoriasis and UVB irradiation [15,42,43]. Keratinocytes are both sources and targets of the pro-inflammatory chemokine IL-8, which contributes to inflammation by playing a role in attracting and activating neutrophils [44]. IL-8 is also upregulated in psoriasis and after UVB irradiation [15,44]. The chemokine RANTES/CCL5 is overexpressed in psoriatic keratinocytes, acts as a chemoattractant for T cells, and activates them [8,14]. In the present study, treatment of TNF-α induced various inflammatory responses including production of pro-inflammatory cytokines IL-6, IL-8 and RANTES in HaCaT cells (Figure 4). Treatment of GEF at 0.1–1 µg/mL attenuated levels of such pro-inflammatory cytokines including TNF-α in keratinocytes stimulated with exogenous TNF-α (Figure 4). Moreover, we also showed that GEF reduced TNF-α and IL-6 release in UVB-irradiated HaCaT cells, indicating the anti-inflammatory effect of GEF (Figure 7). Our findings demonstrate that GEF-mediated suppression of pro-inflammatory cytokine production may contribute to the treatment of skin inflammatory conditions.

IL-10 is an anti-inflammatory cytokine. Keratinocytes are both targets of IL-10 and active producers of this cytokine. Photodynamic treatment stimulates keratinocytes to produce IL-10 [45,46]. Since IL-10 inhibits production of pro-inflammatory cytokines such as IL-1, IL-6, and TNF-α, it can be useful for reducing inflammatory skin conditions, like psoriasis [47,48]. In this study, GEF stimulated production of anti-inflammatory cytokine IL-10 and reduced TNF-α production in TNF-α treated HaCaT cells, supporting anti-inflammatory effect of IL-10 and GEF.

Several anti-inflammatory agents are known to increase antioxidant molecule and anti-inflammatory markers such as IL-10, through activation of the nuclear factor erythroid 2 related factor 2 (Nrf2) pathway in keratinocytes [49]. Previous study has shown that gintonin activates the Nrf2 pathway by promoting its nuclear translocation in cellular and animal model of 3-nitropropionic acid-induced Huntington’s diseases [50]. These anti-inflammatory effects may be associated with the interplay between the Nrf2 and NF-κB pathways.

The transcription factor NF-κB is associated with various cellular events including regulation of inflammatory response, immune modulation, apoptosis, cell proliferation, etc. [51]. In inflammatory responses, NF-κB modulates various cytokines such as TNF-α, IL-1, IL-6, IL-8, RANTES, and macrophage-inflammatory protein-1 [29,51,52,53]. It also modulates inducible enzymes COX-2 and iNOS [15,51]. In the early phase of inflammation, COX-2 and iNOS induce the production of PGE_2_ and nitric oxide, which are major inflammatory mediators [54,55]. NF-κB is involved in elevated expression of COX-2 and iNOS in inflammatory stimuli [15,51,55]. NF-κB was activated and translocated to nucleus in preceded appearance of cytokines IL-6 and RANTES in keratinocytes [56,57]. Nuclear translocation of NF-κB serves as an indirect marker of its activation [56,57]. In this study, GEF (1 µg/mL) partially inhibited NF-κB nuclear translocation in TNF-α-stimulated HaCaT cells, suggesting reduced NF-κB transcriptional activity. This inhibition may underlie the reduced expression of COX-2 and iNOS, and the reduced production of PGE_2_, nitric oxide, IL-6, and RANTES.

In conclusion, in this study, GEF decreased the TNF-α-induced ROS production in HaCaT cells. GEF also reduced the release of pro-inflammatory cytokines including IL-6, TNF-α, and RANTES but increased IL-10 release in TNF-α-treated HaCaT cells. Moreover, treatment with GEF attenuated TNF-α-induced COX-2 expression, PGE_2_ release, and NF-κB translocation in HaCaT cells. Additionally, UVB irradiation-induced pro-inflammatory cytokines TNF-α and IL-6 levels were reduced by GEF treatment in HaCaT cells. These results indicate that GEF may inhibit TNF-α-induced inflammatory responses in keratinocytes, leading to its anti-inflammatory activity in the skin. Therefore, GEF may be a potential natural therapeutic agent for the treatment of inflammatory skin disorders. This study provides a basis for the development of novel therapeutic approaches for the treatment of inflammatory skin disorders.

## 4. Materials and Methods

### 4.1. Materials

GEF was prepared from *P. ginseng* root as described previously [21]. Briefly, 1 kg of 4-year-old ginseng was ground (>3 mm) and extracted with 70% fermented-ethanol eight times for 8 h each time at 80 °C using reflux system [21]. The extract was concentrated, dissolved in cold distilled water (at a ratio of 1:10) and stored at 4 °C for 24 h. The precipitate was lyophilized to obtain GEF (about 1.3% yield) [21]. LPA (LPA 18:1, 1-oleoyl-2-hydroxy-sn-glycero-3-phosphate) was purchased from Avanti Polar Lipids (Alabaster, AL, USA). Anti-COX-2 antibody, anti-iNOS antibody, and anti-β–actin-horse radish peroxidase (HRP) conjugated antibodies were purchased from Abcam (Cambridge, MA, USA). Goat anti-rabbit IgG secondary antibodies were purchased from GeneTex (Irvine, CA, USA). ELISA kits (DuoSet^TM^ ELISA kits) for human IL-6, IL-8, IL-10, TNF-α, and RANTES/CCL5 were purchased from R&D systems (Minneapolis, MN, USA). ELISA kit for PGE_2_ was also purchased from R&D systems (Minneapolis, MN, USA). Dulbecco’s modified Eagle Medium (DMEM) (low glucose) was purchased from Welgene Inc. (Gyeongsan-si, Republic of Korea). Ez-Cytox was purchased from DoGenBio (Seoul, Republic of Korea). Anti-NF-κB p65 polyclonal antibody and all other materials were purchased from Thermo Fisher Scientific Korea (Seoul, Republic of Korea).

### 4.2. Cell Culture

HaCaT human skin keratinocytes were supplied by Prof. H.M. Lee from Hoseo University (Asan-si, Republic of Korea). The cells were maintained in DMEM containing 10% (*v*/*v*) fetal bovine serum (FBS), 100 U/mL penicillin, and 100 μg/mL streptomycin at 37 °C in a humidified atmosphere containing 5% CO_2_.

### 4.3. Cell Viability Assay

Cell viability was assessed using the WST-based assay (EZ-Cytox, DoGenBio) according to the manufacturer’s instructions and previously described methods [32]. Briefly, cells (5 × 10^3^ cells/well) were seeded into 96-well plates, incubated for 48 h, and serum starved for 4 h. These cells were then pretreated with GEF at the indicated concentrations for 1 h. Subsequently, the cells were incubated in the presence or absence of 10 ng/mL of TNF-α for 24 h. LPA (10 µM) was used as a positive control for the cell viability test. The culture medium was replaced with fresh serum-free medium without phenol red. These cells were then treated with the EZ-Cytox solution (DoGenBio). Cell viability was assessed by measuring absorbance at 450 nm using a plate reader (Spectra Max 190, Molecular Devices, Sunnyvale, CA, USA).

### 4.4. ROS Production Assay

The cells were pretreated with GEF at the indicated concentrations for 1 h. Subsequently, the cells were incubated in the presence or absence of 10 ng/mL TNF-α for 40 min. TNF-α-stimulated ROS production in cells was analyzed using the cell-permeable fluorogenic probe, 5-(and-6)-chloromethyl-2′,7′-dihydro-fluorescein diacetate, acetyl ester (CM-H2DCFDA) for 30 min, according to the manufacturer’s instructions (Thermo Fisher Scientific Korea, Seoul, Republic of Korea). Briefly, the cells were incubated with 10 µM of CM-H2DCFDA in phosphate-buffered saline (PBS) followed by washing with PBS. Then, the cells were fixed with 4% paraformaldehyde in PBS and subsequently washed with PBS twice. The fluorescence intensity was measured at an excitation wavelength of 485 nm and emission wavelength of 535 nm using a fluorescence microplate reader (Gemini EM, Molecular Devices, Sunnyvale, CA, USA). Fluorescent images were captured at 100× magnification using an inverted fluorescence microscope (Carl Zeiss, Axiovert 200M; Baden-Württemberg, Germany).

### 4.5. NO Production Assay

NO release was measured using Griess reagent according to the manufacturer’s instructions (Thermo Fisher Scientific Korea, Seoul, Republic of Korea). Briefly, cells (2 × 10^5^ cells/well) were seeded into 6-well plates, incubated for 4 days, and serum-starved for 4 h. These cells were then pretreated with GEF at the indicated concentrations for 1 h and then incubated in the presence or absence of 10 ng/mL of TNF-α for 24 h. The supernatant of the culture medium (conditioned medium) in each well was incubated with Griess reagent (20 mg/mL) for 15 min. The absorbance was measured at 540 nm within 30 min using a Spectra Max 190 plate reader (Molecular Devices, San Jose, CA, USA).

### 4.6. Cytokine and PGE_2_ Analysis

Cytokine levels were measured as previously reported [15] with some modifications. Briefly, HaCaT cells were incubated with serum-free DMEM at 37 °C for 4 h and with DMEM in the presence or absence of GEF at different concentrations for 1 h. Then the cells were incubated in the presence or absence of 10 ng/mL of TNF-α for 24 h. Levels of cytokines (IL-6, IL-8, IL-10, RANTES/CCL5, TNF-α) in the supernatant of cultured medium (conditioned medium) was measured using an ELISA kit (DuoSet^TM^ ELISA kit) (R&D Systems, Minneapolis, MN, USA) according to the manufacturer’s instructions. Parameter^TM^ prostaglandin E_2_ assay kit (R&D Systems, Minneapolis, MN, USA) was used for the determination of PGE_2_ in cell culture supernatants according to the manufacturer’s instructions.

### 4.7. Immunoblotting

The levels of COX-2 and iNOS in cell lysates were analyzed by immunoblotting. Cells were lysed with a modified radioimmunoprecipitation assay buffer containing protease and phosphatase inhibitors. Proteins were separated using sodium dodecyl sulfate-polyacrylamide gel electrophoresis and transferred onto polyvinylidene fluoride membranes. The membranes were incubated with rabbit anti-COX-2 polyclonal antibody (1:1000), anti-iNOS polyclonal antibody (1:1000), or goat anti-rabbit IgG antibody conjugated to HRP. The membranes were stripped and re-probed with HRP-conjugated mouse anti-β-actin monoclonal antibody (1:30,000) as a loading control for the cell lysates. The blots were developed using Clarity Western ECL Substrate (Bio-Rad, Hercules, CA, USA) and images were obtained on an iBright CL1500 imaging system (Thermo Fisher Scientific, Waltham, MA, USA).

### 4.8. Immunostaining

iNOS and NF-κB proteins in cells were detected by immunocytometry. The cells (4 × 10^4^ cells/well) were seeded and grown on glass coverslips coated with collagen in a 12-well plate for 48 h. Cells were pretreated with GEF at the indicated concentrations for 1 h, followed by incubation with or without 10 ng/mL TNF-α for 30 min (NF-κB detection) or 24 h (iNOS detection). The treated cells were fixed with 4% paraformaldehyde in PBS containing 0.1% Triton X-100, washed with PBS, and blocked with 3% bovine serum albumin in PBS for 1 h. Cells on the coverslips were incubated with rabbit anti-NF-κB polyclonal antibody (1:200) or rabbit anti-iNOS monoclonal antibody (1:200) at 4 °C overnight. The cells were then washed with PBS and incubated with a secondary antibody labeled with goat anti-rabbit Alexa Fluor 594 (1:200) for 2 h. After washing, the stained cells were mounted with a mounting medium containing DAPI for nuclear staining and visualized using an inverted fluorescence microscope (Carl Zeiss, Axiovert 200M, Baden-Württemberg, Germany).

### 4.9. UV Irradiation

Cells (1 × 10^4^ cells/well) were seeded into 96-well plates, incubated for 48 h, and serum starved for 2 h. The cells were irradiated with UVB (10 mJ/cm^2^) in PBS using a UVB lamp (312 nm, Super Light-VI, BoTeck, Gunpo-si, Republic of Korea). After UV irradiation, the cells were incubated in a serum-free medium with GEF at the indicated concentrations for 24 h. The media in which the cells were cultured were collected and then centrifuged. Cytokine release was measured in the collected supernatants using ELISA.

### 4.10. Statistical Analysis

Statistical significance was determined using Student’s *t*-test for pairwise comparisons and one-way ANOVA followed by Dunnett’s post hoc test for multiple group comparisons. Data analyses were performed using GraphPad Prism (version 5.0; GraphPad Software Inc., La Jolla, CA, USA). Results are expressed as the mean ± standard error of the mean (S.E.M) from the indicated number of replicates. Statistical significance was set at *p* values < 0.05.

## Data Availability

The original contributions presented in this study are included in the article. Further inquiries can be directed to the corresponding author(s).

## Abbreviations

The following abbreviations are used in this manuscript:

NO	nitric oxide
NF-κB	nuclear factor-κB
iNOS	inducible nitric oxide synthase
IL-6	interleukin-6
IL-8	interleukin-8
IL-10	interleukin-10
RANTES	regulated upon activation, normal T cell expressed and secreted
CCL5	chemokine (c-c motif) ligand 5
GEF	gintonin-enriched fraction
TNF-α	tumor necrosis factor-α
UVB	ultraviolet B
